# Supplementary material for: Effects of Natural Habitat and Season on Cursorial Spider Assemblages in Mediterranean Vineyards
Source: Insects. 2023 Sep 25;14(10):782. doi: 10.3390/insects14100782 (PMC10607350; doi:10.3390/insects14100782)
Supplement: Supplementary file 1 [file insects-14-00782-s001.zip › insects-2604878-Supplementary Table S1 and S2.pdf]

Supplementary Materials:

Supplementary Table 1. Selected vineyard sites in the Qedesh valley, Upper Galilee, Israel, and number of agrochemical applications (H = herbicides; P = pesticides; F = fungicides).

Coordinates	Vineyard	Area (m2)	Perimeter (m)	Elevation (m asl)	Number of agrochemical applications		
					H	P	F
33.131 N, 35.538 E	Yiftah	26075.97	699	425	3	3	5
33.111 N, 35.533 E	Zar'it	16848.89	571	423	2	2	4
33.094 N, 35.528 E	Dishon	34886.65	655	427	2	2	5

Supplementary Table 2. Activity density (spider abundance/ no. of traps * no. of days ± SD) for each spider family (Gna = Gnaphosidae, Zod = Zodariidae, Sal = Salticidae, Lyc = Lycosidae, Phi = Philodromidae, Lin = Linyphiidae) in each of the four positions (NH = natural habitat, BO = border, CE = vineyard center, VV = border between the focal vineyard and the neighboring vineyard), at each vineyard site (Dis = Dishon, Yif = Yiftah, Zar = Zar'it) in May-June and August.

Month	Site	Position	Total	Gna	Zod	Sal	Lyc	Phi	Lin	no. of traps
May-Jun	Dis	NH	32 ± 6.58	6 ± 1.79	8 ± 2.61	3 ± 1.34	5 ± 1.00	1 ± 0.45	0	5
		BO	35 ± 3.24	3 ± 0.89	4 ± 1.30	3 ± 0.89	13 ± 2.07	3 ± 0.55	3 ± 0.89	5
		CE	38 ± 2.94	2 ± 0.82	4 ± 0.52	5 ± 1.33	6 ± 0.89	1 ± 0.41	4 ± 1.21	6
		VV	34 ± 2.17	6 ± 1.10	6 ± 0.45	3 ± 0.55	6 ± 1.64	1 ± 0.45	2 ± 0.55	5
	Yif	NH	56 ± 3.01	14 ± 1.37	7 ± 0.98	11 ± 1.47	3 ± 0.55	4 ± 0.82	2 ± 0.52	6
		BO	31 ± 1.79	6 ± 1.30	2 ± 0.55	1 ± 0.45	6 ± 0.45	1 ± 0.45	3 ± 0.55	5
		CE	29 ± 2.48	4 ± 0.52	1 ± 0.41	4 ± 0.82	3 ± 0.84	5 ± 1.17	6 ± 1.26	6
		VV	16 ± 1.37	1 ± 0.41	0	3 ± 0.55	2 ± 0.52	4 ± 1.21	4 ± 0.82	6
	Zar	NH	20 ± 2.35	7 ± 1.67	1 ± 0.45	3 ± 0.55	2 ± 0.55	4 ± 1.30	2 ± 0.89	5
		BO	16 ± 3.16	4 ± 1.15	1 ± 0.50	1 ± 0.50	1 ± 0.50	2 ± 1.00	1 ± 0.50	4
		CE	18 ± 2.68	1 ± 0.41	0	1 ± 0.41	4 ± 0.82	2 ± 0.82	2 ± 0.82	6
		VV	6 ± 2.00	0	0	1 ± 0.58	2 ± 1.15	0	0	3
Aug	Dis	NH	12	1	4	1	4	0	0	1
		BO	22 ± 2.66	3 ± 0.55	1 ± 0.41	7 ± 1.47	0	2 ± 0.52	5 ± 2.04	6
		CE	22 ± 2.08	1 ± 0.50	5 ± 1.89	9 ± 1.71	3 ± 0.96	1 ± 0.50	0	4
		NV	20 ± 2.12	7 ± 1.67	3 ± 0.89	5 ± 0.71	4 ± 0.84	0	0	5
	Yif	NH	28 ± 7.89	17 ± 5.60	6 ± 1.67	1 ± 0.41	0	2 ± 0.52	0	6
		BO	49 ± 4.17	18 ± 1.26	17 ± 2.14	2 ± 0.52	1 ± 0.41	2 ± 0.82	3 ± 0.55	6
		CE	22 ± 6.02	7 ± 1.83	6 ± 2.45	1 ± 0.41	0	1 ± 0.41	2 ± 0.82	6
		VV	66 ± 4.52	7 ± 0.41	24 ± 3.16	0	2 ± 0.52	12 ± 1.55	5 ± 0.98	6
	Zar	NH	16 ± 1.53	7 ± 1.53	0	3 ± 1.00	2 ± 1.15	1 ± 0.58	0	3
		BO	51 ± 6.77	26 ± 5.35	0	3 ± 0.55	2 ± 0.82	13 ± 2.04	0	6
		CE	17 ± 1.82	4 ± 0.84	0	4 ± 1.10	3 ± 0.89	5 ± 1.00	0	5
		VV	32 ± 2.94	10 ± 1.51	0	8 ± 1.75	2 ± 0.52	6 ± 1.26	2 ± 0.52	6
